# Editorial: Advances in plant-soil nitrogen management strategies

**DOI:** 10.3389/fpls.2024.1380284

**Published:** 2024-02-09

**Authors:** Fahad Shafiq, Sumera Anwar, Shahbaz Khan

**Affiliations:** ^1^ Department of Botany, Government College University Lahore, Lahore, Pakistan; ^2^ Department of Biosciences, Durham University, Durham, United Kingdom; ^3^ Department of Botany, Government College Women University Faisalabad, Faisalabad, Pakistan; ^4^ College of Tropical Crops, Hainan University, Haikou, China

**Keywords:** nitrogen management, cropping patterns, soil health, sustainable agriculture, soil fertility

Life on Earth is impossible without nitrogen (N). It is essentially required for all living organisms to synthesize amino acids, vitamins, proteins, enzymes, co-enzymes, and nucleic acids. This intensive dependence of living cells on N for almost all the metabolic and regulatory processes requires demands N uptake, its transport/mobilization and re-mobilization within plant parts, and synthesis of N storage proteins. The purpose of this focused Research Topic is to improve our understanding regarding different aspects of N mobilization/re-mobilization, influence of the cropping patterns and tillage practices on soil N transformations. Therefore, some key findings from different studies published in this Research Topic are summarized here.

Nitrogen is essentially required by plants for proper growth and development, whereas its deficiency triggers several metabolic and physiological changes in plants. In this context, Effah et al. studied the metabolomic profile of senescent wheat leaves and provided some key insights into N-remobilization. Widely untargeted metabolomic profile of the senescent wheat leaves under no, low, and high nitrogen (N) conditions across three phenological stages (anthesis, grain filling, and end grain filling stages) identified a total of 826 secondary metabolites; most of them were flavonoids and phenolic lipids. The authors concluded that lysine degradation and biosynthesis of alkaloids (primarily from lysine, ornithine, and nicotinic acid pathways) was primarily up-regulated under N deficiency growth conditions. Whereas, the high-N promoted the accumulation of flavones, flavonols, and anthocyanins. Similarly, Song et al. investigated nutrient stoichiometric ratios and resorption efficiency in fresh petals and petal litter of four tree species from the Rosaceae family. A lower nutrient resorption efficiency and stoichiometric ratio of petals compared to leaves was recorded. Moreover, the nutrient resorption efficiency was also strongly correlated with nutrition availability. These findings could provide key insights into N- management in tree species. By contrast, Chen et al. reported that the application of ammonium-nitrate mixtures improved N uptake and assimilation by pecan which substantially improved its growth characteristics. Pecan seedlings were exposed to different NH_4_^+^:NO_3_^-^ sourced N regimes at 0:100; 25:75, 50:50; 75:25, and 100:0 respectively and the data was recorded after 45 days of experimental treatments. An improvement in the root proliferation, relative growth rate (RGR), biochemical traits, and enzymatic activities of N assimilating enzymes viz. nitrate and nitrite reductases; glutamine and glutamate reductases; and glutamate dehydrogenase was recorded. In an overall assessment, these improvements in physio-biochemical traits were particularly evident in response to 75:25 and 100:0 NH_4_^+^:NO_3_^-^mixtures. Huang et al. proposed high-throughput root phenotyping (HTRP) as a tool to identify low-N-tolerant crops under waterlogged conditions. Several key root traits were highlighted in the review, including root length, surface area, and root architecture can be selected and analysed via imaging tools including MIFE, EMI, GPR, MRI, and other techniques including rhizotron imaging and X-ray CT to establish roots 3D structure. The authors also discussed experimental methods to study roto phenotypes and the limitations associated with them. In the final part of the mini-review, various image analysis tools are discussed which can be used to visualize root activity and HTRP and both the advantages and limitations associated with the imaging tools are provided. In summary, the analyses of root system architecture could provide key information in the context of N acquisition from the soil, and HTRP at the field scale could provide a better understanding of root NUE under field conditions.

It is well accepted that cropping patterns play a significant role in soil-N transformations and the soils with multiple cropping patterns improve N-recycling (Breza et al., 2023). Lesser crop rotation frequency ultimately leads to degradation of soil organic matter fraction and thereby contributes to N-losses (Khan et al., 2007). Alternatively, an increase in the number of rotational crops improved crop yields owing to better functional diversity (Bowles et al., 2020). Taken together, cropping patterns do influence N-mobilization and its availability in agricultural soils and studies focusing on cropping patterns will help us understand and enhance soil N status sustainably. By using a radiolabeled ^15^N-isotope, Jia et al. determined the fate of N and its transport in soil under a wheat-rice rotation system. During this study, both N-utilization by crops and residual soil N were investigated in response to fertilizer, soil, and straw respectively. Interestingly, results revealed that a major fraction of crop N was derived from soil, followed by fertilizer and straw (averaging ~ 44.1, 35.9, and 19.0% respectively). However, N loss was prominent from soil, followed by fertilizer and straw application. In addition, soil N contributed to the highest N-utilization efficiency at 38.2%, followed by the contribution of fertilizer at 30.1% and contribution of straw was the least (around 13.9%). Post-seasonal N variations in the soil indicated an average loss of N by 7.3% from soil and 13.8% from fertilizer, whereas the straw contributed to soil N recovery by 4.38%. Above all, it is concluded that the grains exhibited maximum N translocation followed by stem and leaves. Likewise, Zhu et al. studied the influence of different N forms (ammonium and nitrate provided with and without dicyandiamide) on soil health and cucumber productivity during seven growing seasons in a monocropping system. The soil health index (SHI) calculations revealed improvements in soil health due to nitrates (SHI_NN_ and SHI_NN+DCD_), whereas soil health was negatively correlated with plant productivity. It was concluded that the application of nitrate under monoculture could promote soil health. Wang et al. conducted a two-year field experiment to investigate the effects of biochar (0, 15, and 30 t ha^-1^) and varying N levels (135, 180, and 225 kg ha^-1^) on plant productivity under the maize-soybean intercropping system. The maize and soybean plants were cultivated in alternating rows (two rows each) and changes in growth, water use efficiency (WUE), and nitrogen recovery efficiency (NRE) were analyzed. Biochar soil amendment promoted maize starch contents during the second year. In addition, maximum grain yield was recorded in response to biochar application at 15 t ha^-1^; whereas N was found beneficial at ~180 kg ha^-1^ respectively. Nonetheless, the biochar improved grain yield, WUE, and NRE, and combined application of biochar and N was most effective method which could reduce N inputs in an intercropping system. During another two-year experiment, Liu et al. investigated the influence of straw mulching on the growth, physiological, and agronomic characteristics of maize/sorghum intercropping system. Straw mulching was carried out at 0, 4.8, 7.2, and 9.6 t ha^-1^ doses. A significant increase in the photosynthesis rate (*P_n_* rate) of maize by up to 15% was recorded when compared to no-mulching, whereas *P_n_* rate of soybeans was enhanced by 24.5% on average. Moreover, prominent enhancements in N-uptake of both the crops was evident. Above all, the results indicated a 66.6% increase in maize crop yield in response to mulching, however, the mulching under inter-cropping caused a 15.4% yield improvement.

During a study by Yan et al., field experiments involving two cropping systems were performed. The first cropping system was the wheat-rice cropping system which utilized wheat straw mulching; whereas the second cropping system included an oilseed rape-rice cropping system which involved rapeseed straw mulching during the rice growing season. Rice cultivation without mulching served as control (N application at 135 and 48 kg ha^-1^). Furthermore, in a parallel experiment in which ^15^N-labelled urea and straw were applied to investigate N-dynamics and its utilization by plants. An increase in N-uptake especially by oil-seed straw mulching was + 45.1% whereas wheat straw mulching contributed to the enhancement of 9% N-uptake compared to control (fallow-rice rotation). The authors also reported improvement in NUE of crops in response to mulching however a significant amount of fertilizer remains un-utilized showing only 3-4% utilization by rice. It was concluded that straw mulching could effectively promote N-utilization in rice. A four-year field experimentation performed by Wang et al. investigated maize grain and nitrogen use efficiency (NUE) in response to different N-P fertilizer rates under a ridge-furrow rainfall harvesting system (RFRH). The fertilizer treatments included low, medium, and high inputs of fertilizers (N and P_2_O_5_ at 0-0, 150-75, 300-150, and 450-225 kg hm^-2^) respectively. The results indicated a maximum N bioaccumulation in response to medium dose application whereas P uptake was linear and dose dependent. By contrast, both N and P use efficiencies were substantially reduced with an increase in fertilizer dose. The authors concluded that the medium fertilization dose of NP fertilizer at 300-150 kg hm^-2^ resulted in maximum biomass and grain yield. Above all, both NUE and PUE were maximum in response to a lower dose of fertilizer. da Silva et al. investigated residual N release from composted sewage sludge and its influence on crop growth under common bean-soybean-palisade grass crop rotation. The authors also studied changes in N-metabolism, biological N_2_-fixation, and various physiological parameters. Experimentation was performed over two years and results indicated an increase in the crop productivity and biological N_2_-fixation which were linked with regulation of ureide metabolism and enzymatic activities of nitrate reductase, urease, and higher ammonium and nitrate N. Overall, it was concluded that the composted sewage sludge can be used as a residual source of N benefitting crop growth. Whereas Zhou et al. investigated the effects of positional application of N-fertilizer on the growth of maize and root proliferation under a wide-narrow row cropping system. The experimental treatments included both narrow-row and wide-row N applications under high and low N-fertilizer doses respectively. Narrow row combined with higher N rates contributed to increased root growth, surface area, and root density. Whereas wide-row cultivation in combination with high nitrogen significantly improved agronomic efficiency and partial factor productivity of N thereby contributing to improved yield. In short, wide row cropping of maize is suggested as a better approach to improve maize yield.

As per ecological viewpoint, modern agriculture practices have been reliant on intensive N-fertilizer inputs to main soil fertility (Galloway et al., 2008) and this practice has caused many problems and has altered soil-N and microbial dynamics. Huge inputs of N-fertilizers primarily contribute to N losses and pose a threat to terrestrial and aquatic life (Van Grinsven et al., 2013; Breza et al., 2023). Besides, very high levels suppress N mineralization (Mahal et al., 2019), affect soil microbial biodiversity by eutrophication (Liu et al., 2019; Wang et al., 2023), reduce NUE of crops (Sutton et al., 2011; Xu et al., 2020), ammonia volatilization, nitrate leaching, impairment of crop yields (Wang et al., 2015) and contributes of emission of greenhouse gasses (Velazquez et al., 2016). Therefore, understanding the soil N transformations and their recycling in terrestrial ecosystems is necessary (Robertson and Groffman, 2024). In this context, Tian et al. studied the effect of different irrigation levels (75-85%, 65-75%, 55-65%, and 45%-55%) and four N levels (0, 150, 300, and 450 kg ha^-1^) on the distribution of soil nitrate nitrogen (NO_3_^–^N) and water in different soil layers and its effect on wolfberry (*Lycium barbarum* L.). Average soil moisture was maximum at full irrigation and likewise, the N levels were high in response to a high dose of 450 kg ha^-1^. It is also reported that NO_3_^−^–N content in the 0-80 cm soil layer was more sensitive to irrigation. Maximum yield of wolfberry (2623.09 kg ha^−1^) was recorded at 65-75% moisture and kg ha^-1^ N application rate (>18% higher than 75-85% combined with 450 kg ha^-1^). Apart from that, the maximum water consumption was recorded at the flowering stage. The authors concluded that both WUE and NUE were optimum in the ranges of 315.4 to 374.3 mm combined with N at 300.0 to 308.3 kg·ha^−1^ respectively. Yang et al. investigated the influence of soil-applied biochar (0, 600, and 1200 kg ha^-1^) and nitrogen fertilizer (105 and 126 kg ha^-1^) on the enzymatic activities of sucrase, acid phosphatase, and urease enzymes were also determined from the tobacco plants. In addition, soil microbial dynamics including microbial C and N, SOC, soil nitrogen (NO_3_^2-^ and NH_4_^+^), and the influence of biochar/fertilizer application on bacterial communities were also investigated by high throughput amplicon sequencing. The results revealed a dramatic shift in the microbial communities in response to BC and fertilizer application. The dominant bacterial communities included Proteobacteria, Bacteroidetes, Actinobacteria, Firmicutes, and Acidobacteria. Whereas changes in community structure were evident due to high rates of BC and fertilizer application. Apart from this, the application of BC and fertilizer, even at high concentrations benefitted tobacco plants and improved biomass. The authors concluded that the 1200 kg ha^-1 + ^126 kg ha^-1^ application was the best combination. Sarkis et al. quantified the N loss through volatilization when supplied to coffee plantations in the form of ammonia nitrogen (AN), urea (U), and urea with N- (n-butyl) thiophosphoric triamide (UNBPT) at five N doses (0, 150, 275, 400, and 525 kg ha^-1^ year^-1^). Soil microbial biomass, respiration, microbial nitrogen, and enzyme activities of urease, and phosphatase were investigated as well and N levels in different coffee plant parts were also determined. The application of AN nitrogen fertilizer only contributed to 1% N loss whereas for urea fertilizer, losses of around 22%. Additionally, a major fraction of N was localized in the beans and husks of the harvested fruits. Above all, the study provided valuable insights into soil microbial dynamics under different N levels. Lastly, Saha et al. reviewed the mechanisms and factors that control fine root decomposition in forest ecosystems and their involvement in global C and N dynamics. There is a substantial contribution of biotic and abiotic factors to this phenomenon of root decomposition. These include precipitation rates, annual temperature, soil pH, soil salinity, and microbial diversity. Although it is very difficult to study the actual decomposition rates, the authors did report that this phenomenon contributes to the release of both N and P.

## Author contributions

FS: Project administration, Writing – original draft, Writing – review & editing. SA: Project administration, Writing – review & editing, Conceptualization. SK: Project administration, Writing – review & editing, Conceptualization.
